# Quality assessment and control of tissue specific RNA-seq libraries of Drosophila transgenic RNAi models

**DOI:** 10.3389/fgene.2014.00043

**Published:** 2014-03-05

**Authors:** Andreia J. Amaral, Francisco F. Brito, Tamar Chobanyan, Seiko Yoshikawa, Takakazu Yokokura, David Van Vactor, Margarida Gama-Carvalho

**Affiliations:** ^1^Universidade de Lisboa, Faculdade de Ciências, BioFIG-Centre for Biodiversity, Functional and Integrative GenomicsLisbon, Portugal; ^2^Instituto de Medicina Molecular, Faculdade de Medicina, Universidade de LisboaLisbon, Portugal; ^3^Formation and Regulation of Neuronal Connectivity Research Unit, Okinawa Institute of Science and Technology Graduate UniversityOkinawa, Japan; ^4^Department of Cell Biology, Harvard Medical SchoolBoston, USA

**Keywords:** drosophila, RNA-seq, central nervous system, shRNA transgenic strain, brain

## Abstract

RNA-sequencing (RNA-seq) is rapidly emerging as the technology of choice for whole-transcriptome studies. However, RNA-seq is not a bias free technique. It requires large amounts of RNA and library preparation can introduce multiple artifacts, compounded by problems from later stages in the process. Nevertheless, RNA-seq is increasingly used in multiple studies, including the characterization of tissue-specific transcriptomes from invertebrate models of human disease. The generation of samples in this context is complex, involving the establishment of mutant strains and the delicate contamination prone process of dissecting the target tissue. Moreover, in order to achieve the required amount of RNA, multiple samples need to be pooled. Such datasets pose extra challenges due to the large variability that may occur between similar pools, mostly due to the presence of cells from surrounding tissues. Therefore, in addition to standard quality control of RNA-seq data, analytical procedures for control of “biological quality” are critical for successful comparison of gene expression profiles. In this study, the transcriptome of the central nervous system (CNS) of a *Drosophila* transgenic strain with neuronal-specific RNAi of an ubiquitous gene was profiled using RNA-seq. After observing the existence of an unusual variance in our dataset, we showed that the expression profile of a small panel of marker genes, including GAL4 under control of a tissue specific driver, can identify libraries with low levels of contamination from neighboring tissues, enabling the selection of a robust dataset for differential expression analysis. We further analyzed the potential of profiling a complex tissue to identify cell-type specific changes in response to target gene down-regulation. Finally, we showed that trimming 5′ ends of reads decreases nucleotide frequency biases, increasing the coverage of protein coding genes with a potential positive impact in the incurrence of systematic technical errors.

## Introduction

Whole transcriptome profiling can provide fundamental insights into the changes in cellular functions that occur as a consequence of genetic mutations and therefore help unravel the molecular pathways underlying human disease. Over the last few years, the enormous complexity of higher eukaryotic genomes, which encode a multitude of non-coding and tissue-specific transcripts and isoforms has been abundantly reported (Manak et al., 2006; Gan et al., 2010; Graveley et al., 2011). The ability to generate unbiased transcriptome profiles is important not only to understand how genes are organized and regulated but also to identify potential novel, unannotated transcripts and exons, which may be additional targets of mutation in disease states. Such goals can be achieved by using next generation transcriptome sequencing (RNA-seq), which allows for relatively unbiased measurements of expression levels across the entire length of transcripts, whether known or novel, even at low abundance. However, this technique relies upon cDNA sequencing, for which larger amounts of total RNA are still required in comparison with other high-throughput, microarray based approaches (Wang et al., 2009).

The study of human diseases can be significantly enhanced by the use of animal models, which facilitate the extension of mechanistic studies, therapeutic discovery and development processes. Due to the fact that many basic biological, physiological and neurological properties are conserved between mammals and *flies*, and that nearly 75% of human disease-causing genes are believed to have functional homologs in the fly (Reiter et al., 2001), *Drosophila* has been appreciated in recent years as a powerful model to study a wide range of human disorders. In conjunction with an extensive arsenal of available genetic tools, invertebrate genetic models allow for efficient and genome-wide screens to unravel genetic pathways underlying disease phenotypes and identify modifier loci of disease causing mutations (Fortini and Bonini, 2000).

Several human diseases are associated with genetic mutations that cause a partial reduction of the normal levels or activity of endogenous proteins. For example, Spinal Muscular Atrophy (SMA) is a devastating and early onset neurodegenerative disorder caused by loss-of-function (LOF) mutations of *Survival of Motor Neuron* (*SMN1*). *SMN1* encodes the Smn protein, a key component of the SMN complex, which is essential for the assembly of the splicing machinery. Smn is required for the assembly of spliceosomal snRNP complexes and low levels of Smn were shown to result in a strong reduction in the rate of biogenesis of these complexes in patient derived cell lines (Wan et al., 2005). Contrasting with these seemingly ubiquitous functions, SMA patients suffer from loss of mobility due to morphological and functional abnormality of neuromuscular junctions (NMJ), motor-neuron death and muscle degeneration, with severe forms of the disease resulting in infant death. In spite of decades of research into this disease, the cellular pathways underlying motor neuron degeneration are still poorly understood. In agreement with a central role in the gene expression pathway, Smn has been shown to be an essential protein for cell function, with null mutations resulting in cell death. However, unlike all other animal genomes, humans carry two homologous, nearly identical gene copies encoding the Smn protein, *SMN1* and *SMN2.* In contrast with *SMN1,* 75% of *SMN2* transcripts display skipping of exon 7 due to a non-coding nucleotide substitution in this exon, resulting in the synthesis of very low levels of full length Smn protein. Therefore, SMA is caused by a reduction in the levels of Smn protein due to LOF mutations of *SMN1*, with the severity of the disease being modulated by the amount of Smn protein synthesized from *SMN2*, which shows varying copy numbers in the human population (Azzouz et al., 2004). This dose dependence on Smn protein levels is a hallmark of human disease that cannot be mimicked in animal models by null mutants. Since all non-human animals only have one *SMN* gene, null mutations are either inviable or, in the case of *Drosophila*, have an extremely reduced viability thanks to maternal expression of the Smn protein. Recently, *Drosophila* transgenic RNAi *Smn* hypomorphs have been generated that display NMJ defects in an Smn dose-dependent manner (Chang et al., 2008; Sen et al., 2013), mirroring the *SMN2* dosage dependence observed in SMA patients. The milder nature of these hypomorphic models makes them similar to the human disease and thus opens the possibility to explore neuronal specific *SMN*-dependent transcriptome changes.

*Drosophila* transgenic RNAi models have been developed using a binary system that uses the offspring obtained from a cross of two transgenic fly lines. One of the lines contains a ubiquitous or tissue-specific promoter upstream of the yeast Gal4 transcription factor coding sequence. The second line has an integrated copy of an intron-spliced hairpin transcript that produces a double stranded RNA for any gene of interest, fused to the yeast upstream activator sequence (UAS) that is bound by Gal4. The resultant offspring will therefore only down-regulate the human disease gene in a tissue-specific manner, depending on the GAL4 driver used (Sik Lee, 2003).

Given its small size, obtaining tissue specific samples from *Drosophila* involves delicate manipulation procedures, making it difficult to generate samples free from cells of surrounding tissues. Furthermore, performing RNA-seq in order to profile tissue-specific transcriptomes in *Drosophila* presents another additional challenge due to the large amount of RNA required for the technique, which is usually attained by pooling samples from hundreds of individuals. In order to profile the transcriptome of the central nervous system (CNS) in *Drosophila*, transgenic RNAi models of the neurodegenerative disorder SMA, we conducted a pilot study comparing RNA-seq libraries generated from pooled samples obtained from control and hypomorphic *Smn* RNAi knockdown fly strains.

Here we present and discuss the possibility and limitations of using RNA-seq for characterization of changes in the *Drosophila* neuronal transcriptome from CNS samples of a transgenic fly strain with cell-type specific down-regulation of an ubiquitous gene. While conducting our investigation, we observed the existence of an unusual variance of gene expression within each fly strain, most likely caused by the presence of uneven contamination from surrounding tissues. In order to circumvent such effects, we demonstrate that in addition to standard procedures for read quality filtering, other quality control methods must be implemented to discard libraries that do not accurately reflect the transcriptome of the target tissue. Furthermore, we discuss the limitations of unraveling cell type specific changes in gene expression in the context of complex tissue samples and evaluate the effect of trimming 5′ ends of reads, which display nucleotide frequency biases on sampling errors.

## Materials and methods

### Fly strain, tissue preparation, and RNA extraction

Fly strains we used in this study: *w*; *P*{*w*^+mC^=*GAL*4−*elav*.*L*}3 (Bloomington), *w*; *P*{*UAS*−*PWIZ*}15, *w*; *P*{*UAS*−*Smn*^RNAi−C24^} (Chang et al., 2008), and *w*; *Df*(3*L*)*Smn*^X7^, *P*{*UAS*−*Smn*^RNAi−C24^}/TM6B, Dfd-YFP. To obtain animals with intended genotype, *w*; *P*{*w*^+mC^=*GAL*4−*elav*.*L*}3 females were crossed to males of *w*; *P*{*UAS*−*PWIZ*}15 (in text: elavGAL4 or WT), *w*; *P*{*UAS*−*Smn*^RNAi−C24^} (in text: elavGAL4-C24 or C24), or *w*; *Df*(3*L*)*Smn*^X7^, *P*{*UAS*−*Smn*^RNAi−C24^}/TM6B, Dfd-YFP (in text: Smn^X7^/elavGAL4-C24 or X7/C24). Considering the technical limitations associated to the isolation of neuronal cells, i.e., the lack of well-described cell surface markers, the impact of exogenous GFP overexpression in the maintenance of a stable differentiated phenotype (David Van Vactor, pers. commun.) and the difficulty of maintaining the physical integrity and phenotype of these cells upon tissue disruption, an approach based on manual dissection of CNS tissue was considered to be the most appropriate for profiling RNAi-dependent changes in neuronal gene expression. Approximately 200 late third instar larvae were dissected in order to generate one CNS biological replicate of the corresponding genotype in ice cold PBS. Dissected CNS samples were quickly frozen in TriPure Isolation Reagent (Roche Diagnostics GmbH, Mannheim, Germany). After, each ~200 CNS samples were pooled and crude total RNA for each sample was extracted using TriPure Isolation Reagent. The crude RNA extract was treated with rDNase set (Macherey-Nagel GmbH & Co KG, Duren, Germany) to digest contaminated DNA and was, subsequently, cleaned-up with NucleoSpinRNA Clean-up XS kit (Macherey-Nagel GmbH & Co KG, Duren, Germany) to remove impurities. The purified total RNA was quantified using spectrophotometry (NanoDrop; Thermo Scientific, DE, USA) and microfluidic analyzer, Agilent RNA 6000 Nano kit (Agilent Technologies, Waldbronn, Germany). In total we generated, six biological replicates for WT, seven biological replicates for C24 and 4 biological replicates for X7/C24.

### mRNA-seq libraries and sequencing

On average mRNA-libraries were generated from 10 μg of total RNA and prepared using the TruSeq RNA Sample preparation protocol (Illumina, USA). Information regarding all the used kits for library construction, cluster formation and sequencing is listed in Supplementary Table 1. In summary, after two cycles of poly-A selection, RNA was fragmented to an average length of 300 bp and then converted into cDNA by random priming. The cDNA was then converted into a molecular library in order to generate unstranded paired-end mRNA-seq libraries of 100 bp using the HiSeq2000 (Illumina, USA). Data acquisition and processing was performed using CASAVA v1.8.1 or v1.8.2 (Supplemental Table 1). The GEO accession number for the mRNA-seq datasets is GSE54724.

### Read quality filtering and alignment to reference genome

Raw reads were processed using in house developed PERL scripts in order to filter out reads with unknown nucleotides, reads with homopolymers with length larger of equal than 50 nt and reads with an average of the Phred quality scores lower than 30. Remaining reads were aligned to *D. melanogaster* genome assembly build BDGP5 (Flicek et al., 2013) using BWA v0.6.1 (Li and Durbin, 2009) in paired-end mode and allowing 1 mismatch and a single matching position. SAM output was further processed using SAMTools v0.1.16 (Li et al., 2009) in order to remove duplicated reads. Command lines shown in Supplementary File 1. In order to identify Gal4 transcripts, all reads were also mapped to the GAL4 gene (Gene ID 855828; accession NC_001148.4) using BWA v0.6.1 (Li and Durbin, 2009) and the same alignment parameters as previously described. Nucleotide frequencies were evaluated using FastQC v0.1 (Andrews, 2010). Taking into account that *D. melanogaster*'s genome architecture has shorter intergenic regions and smaller introns than *H. sapiens*, BWA was selected to map reads as it allows for gapped alignments and shows higher sensitivity without sacrificing quality when compared to Bowtie [used by TopHat, (Trapnell et al., 2012) the most used tool for RNA-seq read mapping], especially in the case of paired-end reads (Bao et al., 2011; Medina-Medina et al., 2012). Considering the widespread use of TopHat for mapping reads, we nevertheless compared the performance of this tool with BWA for our mRNA-seq libraries that displayed the expected profile in marker gene expression. In comparison with BWA, TopHat mapped an additional 5% of quality approved reads, but the proportion of pairs that were accurately mapped was 20% lower, therefore supporting our choice of aligner (Supplementary Table 2).

### Quantification of gene expression by mRNA-seq and statistical analysis

SAM outputs filtered for duplicated reads were used in order to perform gene counts using the htseq-count function from HTseq framework v 0.5.3p3 (Anders, 2010) in union mode and discarding low quality score alignments (–a 10), using Flybase annotation of gene models release 5.46 as available in Ensembl for genome assembly build BDGP5 (Flicek et al., 2013) (command line shown in Supplementary File1). For assessment of marker genes within library, reads were normalized using the FPKM method (Trapnell et al., 2012) in order to compare our results with tissue specific RNA-seq expression values available from FlyBase (Marygold et al., 2013). In order to perform analysis of differential expression (DE) between phenotypes, read counts were normalized and tested for DE using an error model that uses the negative binomial distribution, with mean linked by local regression to model the null distribution of the count data implemented in DESeq v1.12.0 (Anders and Huber, 2010) package of Bioconductor v2.10 (Gentleman et al., 2004). Clustering analysis was performed using the heatmap function from ggplot package (default parameters) and correlation plots were generated using lattice package in R environment (R Development Core Team, 2011). Significance of overlap between genes lists was tested using the Hypergeometric test (Apostolico et al., 2007) implemented in R v2.15 (R Development Core Team, 2011). Significance of proportions of genes belonging to tissue-specific or ubiquituous expression groups was tested using the two-proportion *z*-test in R (R Development Core Team, 2011).

### Quantification of Smn expression by RT-qPCR

Total RNA was extracted from CNS samples using the TriPure RNA extraction reagent. RNA samples were treated with rDNase set followed by purification with NucleoSpin RNA Clean-up XS kit. 1 μg of total RNA was reverse transcribed using the PrimeScript® RT reagent Kit (TaKaRa Bio Inc., Shiga, Japan) by following the protocol for TaqMan Probe Assays. qPCR was performed using Premix Ex Taq™ (TaKaRa Bio Inc.) with 100 ng of cDNA in a ABI7500 Real time PCR system (Applied Biosystems). TBP primers and Zen™ double quenched probe sets were designed using the PrimerQuest primer design tool provided by Integrated DNA Technologies, Inc. (Coralville, IA, USA) and synthesized by the same company. Primers for *Smn* quantification were designed and synthesized by TaKaRa Bio Inc. (Shiga, Japan). Primer and probe sequences are presented in Supplementary Table 3. The fold change of the gene expression was determined by relative quantification using comparative Ct.

## Results

### Detailed quality assessment of mRNA-seq libraries identifies biases introduced at the sample isolation and library preparation steps

A *Drosophila* transgenic strain for neuronal specific downregulation of *Smn* expression by double-stranded RNAi (*Smn*^wt^/elavGAL4-C24) was engineered by integrating a GAL4 inducible anti-*Smn* shRNA construct into an *Smn*^wt^/elavGAL4 background (Chang et al., 2008). The *elav* promotor is a post-mitotic neuron-specific driver, allowing for a tissue specific down-regulation of *Smn*, with the aim of characterizing *Smn*-dependent changes in neuronal gene expression. The CNS of late third instar larvae, consisting of optic lobe, central brain and nerve cord, was dissected from 200 animals from *Smn*^wt^/elavGAL4 (WT) and *Smn*^wt^/elavGAL4-C24 (C24) backgrounds and total RNA was isolated and pooled to generate a pilot RNA-seq dataset for WT and C24 strains (composed of three and four replicate pools for each, respectively). Following poly-A RNA enrichment, paired-end RNA-seq (mRNA-seq) libraries with ~100 million reads were generated from the pooled samples (Table 1). In order to remove reads with poor sequencing quality (see Materials and Methods), quality filters were applied. As shown in Table 1, about 80% of reads passed these filters and proceeded for alignment to the reference genome and approximately 90% of these aligned to the *D. melanogaster* genome. However, after removing duplicate reads with potential origin in PCR errors, only between 27 and 60% of the aligned reads remained for further analysis. Excluding the C24_4 library, we found a very strong correlation between the number of duplicates and the amount of cDNA used for library generation (Supplementary Figure 1). After removal of duplicates, the remaining reads that were uniquely mapped to the reference genome were annotated by comparing genomic coordinates with the flybase annotation 5.46 (Marygold et al., 2013). Results shown that on average 75% of reads mapped to protein coding genes, corresponding approximately to 12,000 different genes with a normalized read depth of 1800 reads/gene, from a total of 13,937 annotated protein coding genes. A small proportion of transcripts (1%) was mapped to non-coding RNAs (data not shown). This dataset was estimated to correspond to 90% of the length of the predicted *Drosophila* transcriptome and to a sequencing depth of ~500-fold coverage per strain.

**Table 1:** **Quantification of cDNA input, read quality filtering, mapping and protein coding annotation statistics of paired-end mRNA-seq libraries**.

**Genotype**	**Sample**	**cDNA amount (ng)**	**Total raw reads (millions)**	**Uniquely aligned reads (millions)**	**Uniquely aligned reads without duplicates (millions/%)**	**Reads mapped to genes (millions/%)**	**Number of gene species**	**Normalized average read depth/gene**
**PILOT SEQUENCING ROUND**								
WT	1	5	104.46	85.35	38.04/44.57	29.42/77	11,985	1188
	2	2	106.03	87.69	23.52/26.82	18.04/76.7	10,758	2186
	3	4	112.71	87.02	45.95/52.80	33.83/74	11,712	1273
C24	1	2	95.62	78.47	21.68/27.63	15.77/72.73	10,725	2461
	2	6	112.11	87.95	53.45/60.77	40.19/75	11,632	1353
	3	4	97.43	80.90	31.26/38.64	21.85/70	10,975	1449
	4	3	111.77	85.32	51.07/59.86	38.14/75	12,092	1322
**SECOND SEQUENCING ROUND**								
WT	4	11	126.82	95.5	70.45/73.77	53.95/77	11,964	1442
	5	10	84.38	66.12	47.21/71.40	36.52/77	11,691	1426
	6	12	113.96	87.50	67.27/76.88	53.40/79	11,984	1457
C24	5	13	86.25	65.83	48.39/73.51	36.87/76	11,667	1506
	6	7	100.34	77.9	53.20/68.29	40.47/76	11,669	1311
	7	12	98.35	77.40	53.17/68.70	39.95/75	11,970	1471
**THIRD SEQUENCING ROUND**								
X7/C24	1	4	75.45	60.86	44.83/73.66	33.60/75	11,665	1523
	2	7	95.06	73.81	58.75/79.60	43.81/75	11,935	1473
	3	6	116.27	96.55	73.98/76.62	56.70/77	12,096	1430
	4	3	116.12	88.27	44.06/49.92	33.03/75	11,386	1346

**Figure 1:**
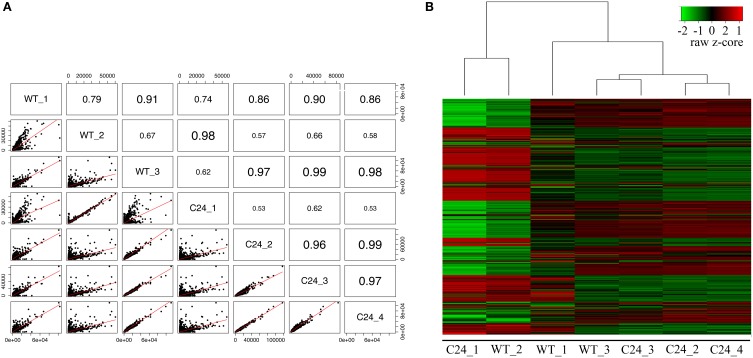
**Transcriptome profiling of *Smn*^wt^/elavGAL4 (WT) and *Smn*^wt^/elavGAL4-C24 (C24) Drosophila strains. (A)** Correlation between samples estimated from normalized counts of gene expression of ~12,000 genes in WT (*N* = 3) and C24 (*N* = 4) paired-end mRNA-seq libraries. Font size is varying according to the value of correlation. **(B)** Overview of hierarchical clustering of mRNA-seq datasets. Hierarchically clustered gene expression profiles were performed using the estimation of stabilized variance of normalized counts of gene expression as in **(A)**. Genes are ordered on the vertical axis. Values are displayed as a Z-score (mean = 0 and *SD* = 1) across samples. The magnitude of deviation from the median is represented by the color saturation. We observed that samples do not cluster according to the known biological origin.

In order to investigate the assignment of each library to its known biological origin, we estimated their degree of correlation (Figure 1A). We observed that WT libraries displayed low correlation with each other, while showing unexpectedly high correlations with specific C24 libraries. The same effect was observed after performing a hierarchical clustering of the stabilized variances (Figure 1B). The clustering analysis grouped libraries into two separate branches, one of which contained the C24_1 and WT_2 libraries, presenting a totally distinct expression profile. Given the technical complexity underlying the collection of these biological samples, we hypothesized that a variable degree of contamination from neighboring tissue might be the main cause of the observed discrepancies.

### Use of marker genes to validate tissue-specificity of mRNA-seq libraries

**Figure 2:**
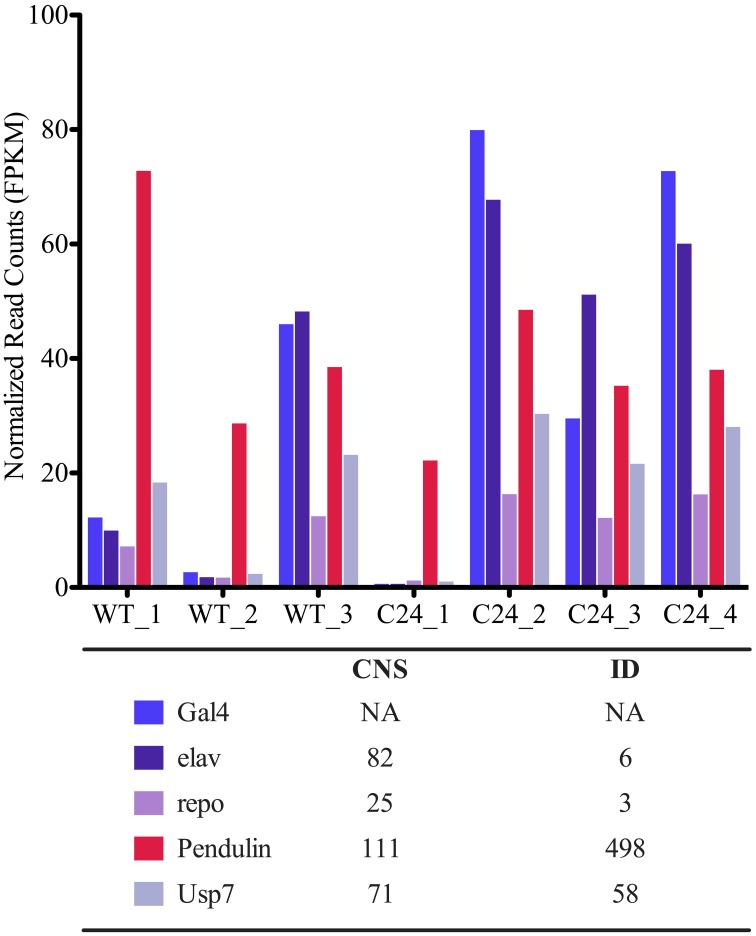
**Assessment of tissue specificity of mRNA-seq libraries using markers genes.** Expression levels of selected marker genes in pilot mRNA-seq libraries using normalized expression counts for gene length (FPKM) (see Materials and Methods). Plot legend presents mRNA-seq FPKM estimates for endogenous genes in L3 stage larvae central nervous system (CNS) and Imaginal Discs (ID) reported by Marygold et al. (2013).

We have hypothesized that the poor clustering of samples according to biological origin resulted from the presence of contamination from close neighboring tissues, such as fragments of imaginal discs (ID), which could have been inadvertently introduced during the delicate step of dissecting hundreds of larvae. In order to test this hypothesis, we defined a panel of five marker genes to which we could confidently assign an expected CNS expression pattern in our fly strains. These included the *GAL4* transgene and the neuronal specific gene *elav,* both displaying neuronal specific expression, the CNS glial cell specific marker *repo* (Xiong et al., 1994), and the *Pen* and *Usp7* genes, which according to published records (Marygold et al., 2013), are not expected to be enriched in the CNS. *Pen* is most highly expressed in ID, whereas *Usp* is a ubiquitously expressed gene. The normalized expression values for gene length (FPKM) for each of these genes were determined for each mRNA-seq library (Figure 2). The results obtained were in agreement with our hypothesis of tissue contamination, with four libraries displaying expected profiles for CNS derived samples, whereas libraries WT_1, WT_2, and C24_1 had high levels of *Pen* expression and low or absent expression of the CNS marker genes. This marker gene approach corroborated the results obtained by the hierarchical clustering analysis and allowed us to identify which expression profile corresponds to the target tissue, thereby providing a biological basis for the exclusion of specific samples from downstream analysis. Although displaying most of the hallmarks of neuronal expression, samples C24_3 and WT_3 presented a lower expression value of the *GAL4* transgene, which suggested that they should not be considered for further analysis. We therefore established a basic profile for CNS expression in our transgenic fly strains, exemplified by the C24_2 and C24_4 samples, which can be used to evaluate the “tissue specificity” of CNS mRNA-seq libraries.

**Figure 3:**
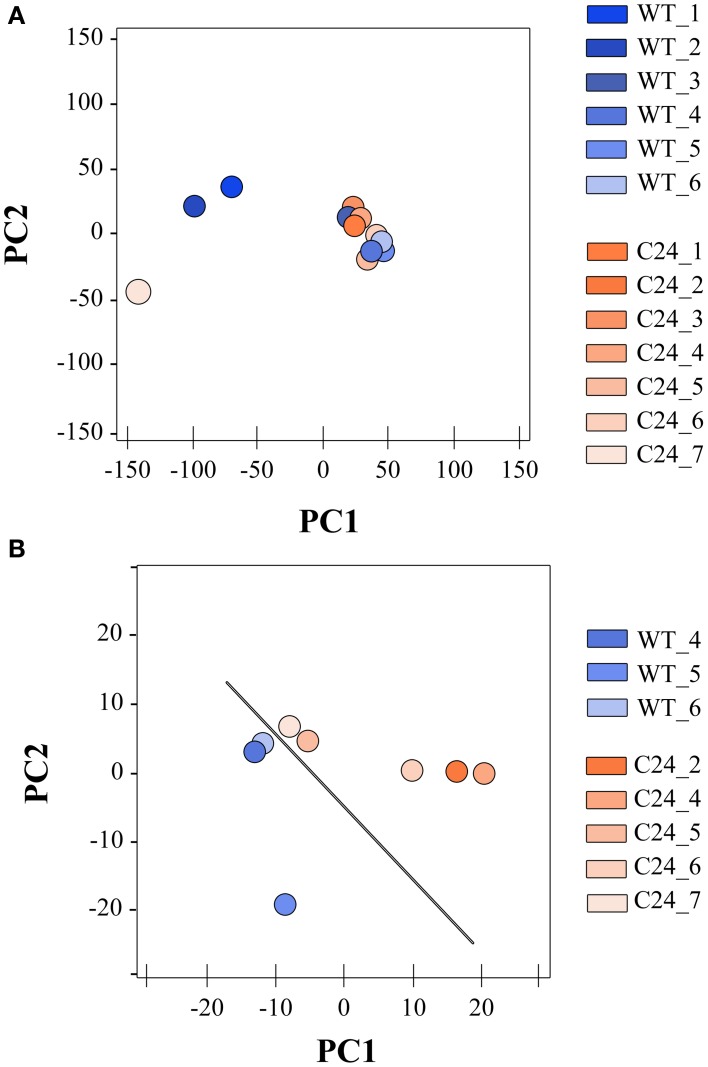
**Marker genes allow identification of mRNA-seq libraries displaying significant contamination from neighboring tissues. (A)** PCA of global expression trends when including all WT and C24 libraries. **(B)** Spatial distribution of global expression trends in WT and C24 excluding libraries flagged by the marker gene panel as displaying significant contamination from neighboring tissues. PCA was applied to the stabilized variances estimated from normalized reads counts of ~12,000 genes expressed in WT and C24 samples.

After this pilot sequencing experiment, six additional paired-end mRNA-seq libraries, corresponding to three biological replicates of WT and C24 pooled CNS samples were generated using higher amounts of input cDNA. This reduced the PCR incurred bias during library preparation and consequently resulted in a higher coverage of protein coding genes, increasing the robustness of the dataset to study the CNS transcriptome (Table 1). We next tested the tissue specificity of the libraries using our gene marker's set. All the novel libraries displayed the expected gene signature for CNS samples of elavGAL4 transgenic flies (Supplementary Figure 2). To evaluate the efficiency of our marker gene panel in detecting non-tissue-specific datasets, we performed a principal component analysis (PCA) including all the sequenced libraries (Figure 3A) or including only the benchmarked libraries (Figure 3B). After removing the potential non-CNS libraries, the PCA analysis displayed a discrete separation between the WT and C24 strains, which was not observed before.

### Sensitivity of detection of cell type specific changes in the expression of ubiquitous genes is limited in complex tissue samples

**Figure 4:**
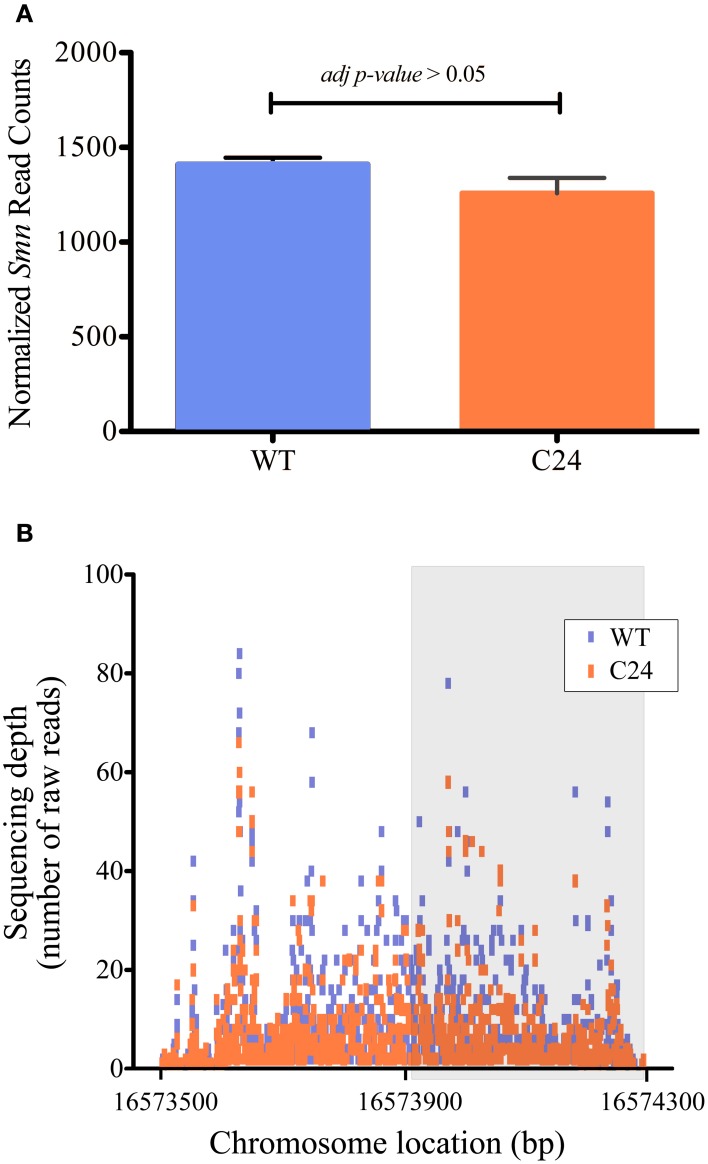
**Quantification of the expression levels of the RNAi target *Smn* in WT and C24 datasets is not affected by detection of reads from the shRNA construct. (A)** Normalized read counts of the *Smn* gene using size factor normalization (see Materials and Methods). Testing for differential expression shows that *Smn* is not significantly down-regulated in the C24 strain (*adjp*-value > 0.05). **(B)** Number of raw sequencing reads aligned along the *Smn* gene in WT and C24 libraries. Gray box marks the *Smn* region targeted by the shRNA transcript. No significant difference between the two strains is observed, suggesting that endogenous gene quantification is not biased by the detection of shRNA derived transcripts.

Following the selection of mRNA-seq libraries to identify datasets displaying low levels of non-CNS tissue contamination, we used libraries from the second sequencing round to perform a comparative analysis between the WT and C24 strains. The results of quantification of gene expression that are presented are based in the BWA alignments that produced a higher accuracy in the mapping of pairs of reads resulting in a higher proportion of reads mapped to protein coding regions. Although the elav-C24 flies exhibit a *Smn*-dependent NMJ phenotype (Chang et al., 2008), the DE analysis identified only a small number of genes (80) when performing a 3 vs. 3 replicate between WT and C24 comparison, of which approximately half were either up or down-regulated. When excluding samples WT_5 and C24_6 and therefore comparing only 2 replicates of each condition, we observed an decrease of the dispersion of measurements of gene expression (Figure 3B), and the number of differentially expressed genes identified increased to 209, of which 141 were up-regulated and 68 were down-regulated in C24 samples. The origin of the elevated dispersion caused by these libraries within their corresponding dataset seems to stem from an unusual presence of transcripts derived from non-CNS tissues, although of a relatively small magnitude (Supplementary Figure 3). Unexpectedly, the list of differentially expressed genes did not include the shRNA target *Smn* as significantly down-regulated (Figure 4A).

The transgenic shRNA knockdown of *Smn* in C24 flies is mediated by the expression of a double stranded RNA corresponding to the 3′ half of the *Smn* transcript (see Figure 4B). Considering the lack of strand specificity of the standard mRNA-seq approach used in this study, it is possible that detection of sequencing reads from the shRNA transgene could bias the overall quantification of the endogenous target gene. To our knowledge, this issue has not been previously addressed in RNA-seq datasets. To evaluate if this transcript was being detected, we mapped the reads that are assigned to *Smn* along its genomic coordinates (Figure 4B). In case of detection of the transgenic shRNA transcript, a marked difference in the number of reads mapping to the targeted region should be observed. Our results indicate that this is not the case. As an alternative approach, we performed a DE analysis of *Smn* levels considering only reads aligning to the non-targeted 5′ region, as the only possible origin for these sequences is the endogenous gene. Therefore these sequence tags will reflect *Smn* expression without the possible confounding effect emerging from the detection of reads from the shRNA transgene. The resulting quantification of *Smn* expression levels was similar to the one obtained using sequencing tags mapping to the full transcript (data not shown), supporting the initial conclusion that *Smn* is not differentially expressed between WT and C24 CNS mRNA-seq libraries. These results suggest that the expression of a transgenic shRNA transcript does not interfere with target gene quantification using non-strand specific, polyA plus paired-end mRNA-seq.

**Figure 5:**
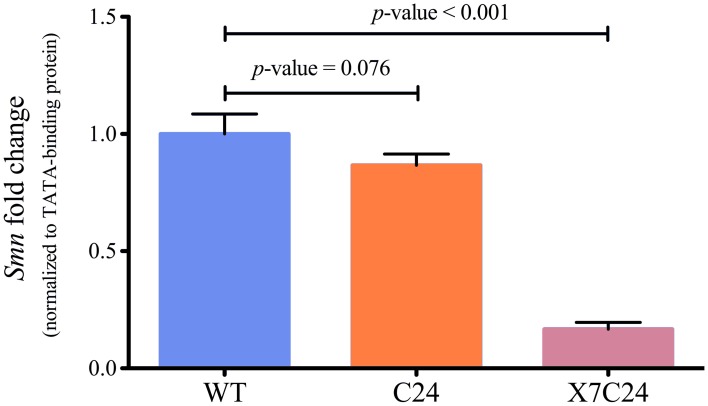
**RT-qPCR quantification of *Smn* expression levels in CNS samples from WT, C24, and X7/C24 fly strains.** Average fold change and standard deviation (*N* = 3). Relative expression levels determined with the 2^∧^ddCt method using the *TATA-Binding protein* as reference gene. *Smn* is only significantly down regulated in *Smn*^X7^/elavGAL4-C24 (X7/C24) flies (*t*-test *p*-value < 0.0001).

**Figure 6:**
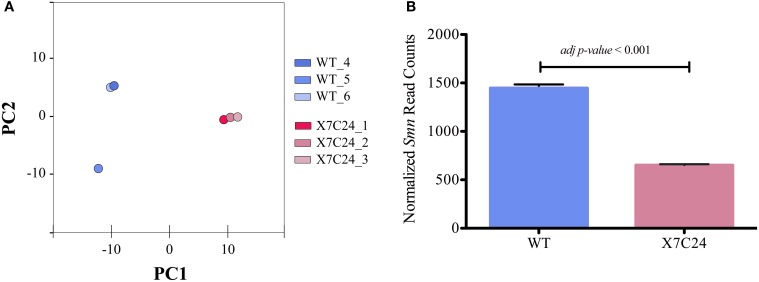
**Comparative analysis of global and *Smn* expression profiles in *Smn*^X7^/elavGAL4-C24 (X7/C24) and elavGAL4 (WT) flies. (A)** Spatial distribution of global expression trends in WT and X7/C24. PCA was applied to the stabilized variances estimated from normalized reads counts of ~12,000 genes expressed in WT and X7/C24 samples. **(B)** Normalized reads counts of *Smn* gene in WT and X7/C24 phenotypes. The transgenic X7/C24 presents a transcriptome profile that clearly distinguishes it from the WT and displays a significant down-regulation of the siRNA target gene *Smn*.

In order to obtain an independent estimate of *Smn* expression levels in WT and C24 CNS, RT-qPCR quantification in total RNA samples was performed (Figure 5). The results obtained show a slight, yet non-significant downregulation of *Smn*, in good agreement with the quantification derived from the mRNA-seq analysis. Considering that the C24 shRNA transgene has been previously shown to efficiently down-regulate *Smn* expression when under the control of a ubiquitous driver, and that *Smn*-dependent phenotypic changes have been demonstrated for the elavGAL4-C24 flies (Chang et al., 2008), the inability to detect differential *Smn* expression may stem from a lack of sensitivity to neuronal specific changes of an ubiquitously expressed gene in the context of CNS samples. Indeed, in addition to neuronal cells where the anti-*Smn* shRNA transgene is active, these samples also contain significant amounts of glial cells, which express high levels of Smn and other ubiquitous genes. To obtain a deeper insight into these issues, mRNA-seq libraries were prepared and sequenced from samples obtained from elavGAL4_C24 transgenic flies in a heterozygous *Smn* deficiency (*Smn*^X7^) background (Chang et al., 2008). The X7/C24 flies present a genomic deletion of *Smn*, thereby affecting *Smn* expression across all tissues. In agreement with this, RT-qPCR analysis of *Smn* levels in the X7/C24 strain showed a reduction of mRNA abundance of approximately 80% (Figure 5). These libraries were processed for quality filtering (Table 1) and its tissue-specificity was assessed as previously described (Supplementary Figure 2). From all X7/C24 libraries, only one was removed due to high level of duplicates, and all passed the benchmark controls for low non-CNS tissue contamination. As shown in Figure 6A, the six selected WT and X7/C24 libraries are spatially separated by phenotype by PCA, confirming a robust assignment of sample origin. Importantly, the increased dispersion previously observed within the WT libraries was found to have a small impact on this dataset, as performing PCA without library WT_5 did not affect the degree of spatial separation between WT and X7/C24 (data not shown). Therefore, this library was included in the following step of DE analysis, which identified a total of 2846 differentially expressed genes, including a significant down-regulation of *Smn* in the X7/C24 strain of approximately 70% (Figure 6B). This is in good agreement with the qPCR quantification data (Figure 5). These genes included 90% of the 181 DE genes in the C24 vs. WT comparison, representing a statistically significant overlap (*p*-value < 0.05) between both datasets. This implies that the elav-C24 strain displays *Smn*-dependent changes in neuronal gene expression. We reasoned that DE of *Smn*-dependent ubiquitous genes might be difficult to detect in the elav-C24 background due to signal dilution in a complex sample. On the other hand, it is predicted that the sensitivity to detect changes in the expression of neuronal specific genes should not be impaired or even display a relative higher efficiency in this mutant background that in the X7/C24 flies. In order to test this hypothesis, we retrieved from Flybase (Marygold et al., 2013) all genes annotated as “neuron,” “glia” and “ubiquitous” regarding their expression and used these lists to classify the DE genes identified in the C24 and X7/C24 backgrounds. As shown in Table 2, in agreement with our predictions, a much smaller proportion of ubiquitous genes was identified as differentially expressed in C24 flies than in the X7/C24, when compared to WT flies. Conversely, the C24 background had a higher sensitivity in the identification of neuronal specific changes in gene expression than the X7/C24, even though the overall down-regulation of *Smn* expression in these flies is expected to be higher.

**Table 2:** **Differentially expressed genes in C24 or X7/C24 flies that are classified as having neuronal, glial or ubiquitous expression in Flybase**.

	**Neuronal (442 genes)**	**Glial (74 genes)**	**Ubiquitous (2649 genes)**
C24	20 genes^†^	1 gene	39 genes^†^
(209 DE genes)	(9.5%)	(0.5%)	(18.5%)
X7/C24	109 genes	29 genes	743 genes
(2846 DE genes)	(3.8%)	(1.0%)	(26.1%)

† Statistically significant differences in the relative proportion of identified genes between the two sample types using the two-proportion z-test are highlighted with (p < 0.05%).

We conclude that the major limitation to the detection of neuronal-specific gene expression changes in C24 mRNA-seq CNS-libraries most likely arises from a signal dilution effect imposed by the presence of other cell types where *Smn* expression is normal.

### Trimming 5′ ends decreases bias in nucleotide frequencies and increases gene coverage

**Figure 7:**
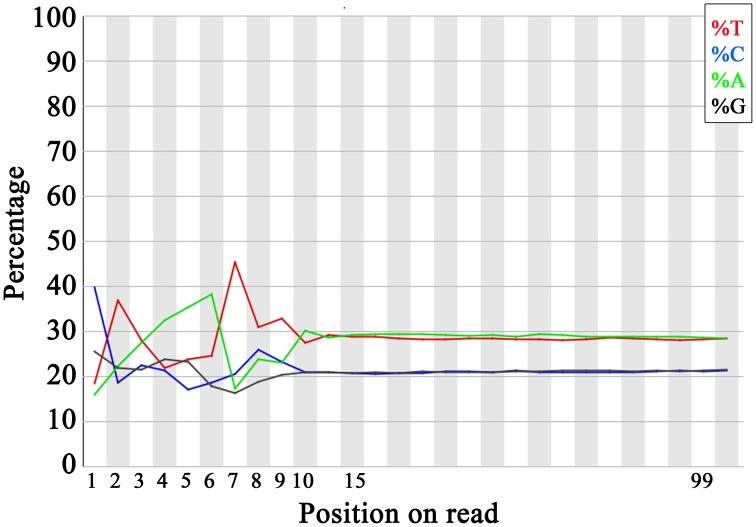
**Bias in nucleotide frequency in 5′ end of reads.** Nucleotide frequencies per position. Example of the first 200,000 reads from the first paired-end sequencing of WT_1, as calculated by the FastQC algorithm (Andrews, 2010). This bias was observed in all the sequenced samples from both paired-end sequencing rounds (data not shown).

**Figure 8:**
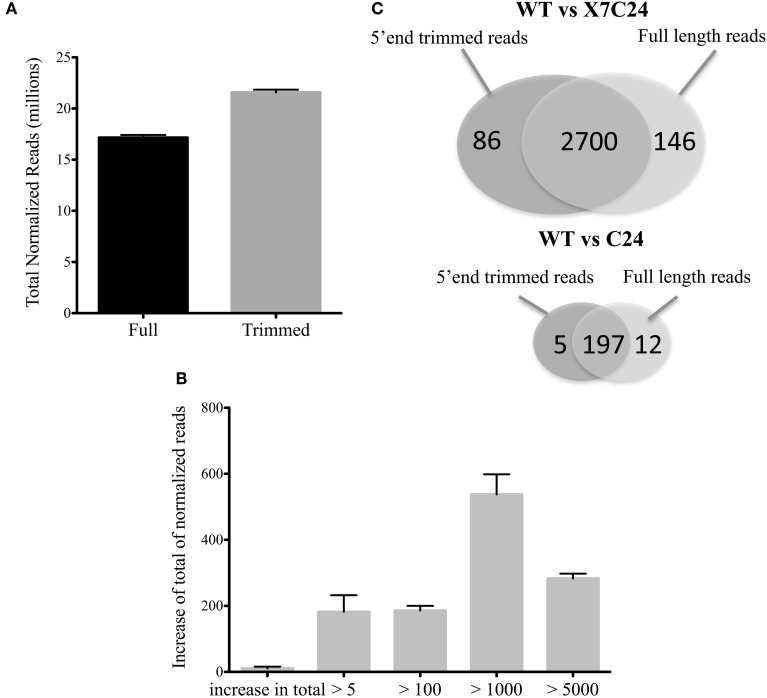
**Effect of 5′ end trimming on gene coverage. (A)** Total reads mapped to protein coding features after performing trimming of 10 nucleotides from 5′ ends of all raw reads (Trimmed) and without trimming (Full) in all libraries of WT, C24, and X7/C24 that passed QC analysis. **(B)** Average gain and s.e.m of normalized reads mapped to protein coding genes after trimming, shown according to class of sequencing depth (estimated over normalized reads) per library (WT, C24, X7/C24). Class of sequencing depth as follows: over 5 reads (>5), over 100 reads (>100), over 1000 reads (>1000), or over 5000 reads (>5000). **(C)** Venn diagram of total number of differentially expressed genes (*adj p-value* < 0.05) in Trimmed vs. Full datasets for WT vs. C24 and WT vs. X7/C24.

Similar to what has been reported in previous RNA-seq studies (Hansen et al., 2010; Jiang et al., 2011), we observed in our dataset a strong distinct pattern in the nucleotide frequencies in the first 10 positions of the 5′ end of sequencing reads (while considering all the raw, unfiltered reads) (Figure 7). This effect reflects a bias in nucleotide content that may consequently cause a decrease in the number reads that are significantly mapped to the reference genome and therefore impact on the quantification of gene expression and/or of transcript isoforms. To our knowledge, in spite of several reports on this effect, no previous study has investigated the impact of these nucleotide biases on the downstream analysis of mRNA-seq libraries. To explore the impact of these biases on sequencing coverage, we trimmed the first 10 nucleotides off the 5′ end of all reads, followed by read quality filtering and alignment to the reference genome as previously described. After filtering, we observed a decrease in library size (mostly due to a decrease of reads with an average quality score larger than 30), accompanied by a larger increase of the number of reads that mapped to the reference genome (data not shown). This resulted in a comparable increase of ~8% of reads that mapped to protein coding genes in all libraries (Figure 8A). This increase in coverage of protein coding genes had larger impact on genes with medium expression levels (Figure 8B), also affecting the DE analysis. The analysis of DE comparing either WT vs. X7/C24 or WT vs. C24 revealed a small decrease in the total number of DE genes and a change in significance affecting 9% of DE genes (Figure 8C).

## Discussion

We have generated the first map of the transcriptome of the CNS of *Drosophila elavGAL4* transgenic strains displaying neuronal specific expression of an shRNA construct targeting the ubiquitously expressed gene *Smn* using paired-end mRNA-seq. The ambitious potential of this experiment is illustrated by the extensive nature of this dataset, with an average of 100 million raw reads per sample, producing a total of 1753 million reads, with potential to cover 95% of the transcriptome and detect the expression of ~12000 genes. However, RNA-seq is not a bias free and 100% accurate approach to obtain a snapshot of the transcriptome in the target biological context (Hansen et al., 2010; Kircher et al., 2011; Sendler et al., 2011). Before sequencing, the generation of mRNA-seq libraries involves processing the extracted total RNA through several steps that are prone to introduce a bias in the accurate measurement of gene and isoform expression. cDNA synthesis followed by PCR amplification for the generation of double stranded DNA is one of the most critical steps in the process and the one with larger impact on the accuracy of transcriptome representation (Ozsolak et al., 2009). Therefore, in order to fully capitalize on the potential of the data generated and extract the maximum amount of biological information during downstream analysis, quality filtering of mRNA-seq is a crucial and important step in the process.

The results obtained in our study using the pilot sequencing run shown that in spite of the fact that only <1% of the raw reads were removed by filtering due to poor quality and that the rate of uniquely aligned reads to the genome was ~90%, the presence of PCR duplicates accounted for an average of 50% of the dataset. This percentage was even higher in datasets in which libraries were prepared from low cDNA inputs, which incurred in a higher rate of PCR errors, as previously reported (Kozarewa et al., 2009). In the libraries generated in the following sequencing rounds, there was an increase in the amount of cDNA used and consequently, we observed the expected decrease in the proportion of duplicated reads. These results clearly demonstrate how the process of mRNA-seq libraries preparation is a critical step that can significantly impact the gene coverage that can be achieved. After duplicate removal, ~90% of quality approved reads were uniquely aligned to the *Drosophila* genome covering at least 90% of the *known* transcriptome, with a sequencing depth of ~500× per gene in each of the studied phenotypes. This level of coverage is of similar magnitude to a previous mRNA-seq study focused on *Drosophila* brain (Hughes et al., 2012) and of similar magnitude to studies focusing on “whole-body” (Gan et al., 2010; Daines et al., 2011), underscoring the potential of this dataset to provide interesting insights on the CNS impact of *Smn* down-regulation and to detect novel transcript isoforms. Similar to a previous study of the transcriptome of the *Drosophila* brain using mRNA-seq (Hughes et al., 2012), our dataset contains only 70% of sequenced tags mapping to protein coding genes, which is 20% less than the reports of Graveley et al. (2011), a previous study which profiled the *Drosophila* transcriptome by mRNA-seq generated from “whole-body” samples. This suggests that the *Drosophila* CNS transcriptome may potentially be enriched with intergenic transcripts, as has been reported in primates (Xu et al., 2010).

As part of the general workflow of bioinformatics analysis after data filtering, the correlation and spatial relation between samples in our dataset was investigated. We observed poor correlations (<0.90) between libraries of the same strain and consequently observed a spurious hierarchical clustering regarding the biological origin of libraries in our pilot sequencing round. Considering the strong correlations observed between some libraries of different biological origin (over 0.98 in some cases), we put forth the possibility that the mRNA-seq libraries were being biased by contaminations from non-CNS tissues originating during the complex step of larvae dissection and tissue isolation, rather than reflecting stochastic biases occurring during library preparation, as reported by others (Mamanova et al., 2010). Therefore, taking into account that our libraries were generated from the CNS of transgenic flies expressing GAL4 under the control of the promoter of the neuronal specific gene *elav*, and that ID are the tissue with highest potential to be unintentionally sampled due to physical proximity, we decided to compare within each sample the levels of expression of: (a) *elav*; (b) the *GAL4* transgene; (c) the glia specific gene *repo* (Xiong et al., 1994); (d) *pendulin*, a gene highly expressed in ID (Marygold et al., 2013) and (d) *Usp*, an ubiquitously expressed gene (Marygold et al., 2013). This small group of genes allowed us to establish a basic profile for CNS-specific expression in our mRNA-seq libraries, which was used to efficiently eliminate datasets displaying large magnitude of contamination from surrounding tissues that compromised DE analysis. This approach is easily adaptable to similar studies using flies that express transgenes from a tissue specific driver, providing a simple solution for the quality control of tissue specific datasets. Following the establishment of this filtering procedure, we set out to evaluate the potential of mRNA-seq analysis of CNS samples to identify differentially expressed genes in response to the neuronal specific down-regulation of our gene of interest *Smn*. The inability to detect a significant down-regulation of the shRNA-targeted gene raised several important questions regarding transcriptome profiling studies in this kind of models. First, we were able to show that although these transgenic flies express high levels of a double stranded RNA that is identical to half of the sequence of the target gene, and in spite of the use of a non-strand specific sequencing approach, we do not detect a significant amount of sequencing reads that may be attributed to the shRNA transgene. Therefore, we conclude that these sequences do not accumulate to levels that may interfere with the quantification of the target gene expression using mRNA-seq. In agreement with this, the results from relative RT-qPCR quantification of the levels of target gene expression were very similar to those determined by mRNA-seq. Therefore, another possible explanation was that either the levels of neuronal-specific down-regulation achieved by the *elav* driver were too low to be detected, or the quantification of transcripts derived from neurons was strongly compromised by the complexity of the tissue sample used to generate the mRNA-seq libraries. The identification of a very small number of differentially expressed genes further raised questions about the relevance of using the *elav*-driven down-regulation of *Smn* to characterize neuronal specific gene expression changes. In order to obtain a deeper insight into these questions, we generated CNS mRNA-seq libraries from progeny obtained by a cross between the elav-C24 line and a Df(3L)SmnX7/TM6B, Dfd, YFP (X7(±)) mutant line, which contains a deletion of the *Smn* gene. These flies have a somatic down-regulation of *Smn*, compounded by the neuronal specific-expression of the anti-*Smn* shRNA and therefore exhibit a much more severe phenotype. In agreement with this, *Smn* down-regulation was very prominent in these flies and a much higher number of genes was found to be differentially expressed when compared to the WT background. Moreover, this group of differentially expressed genes showed a highly significant overlap with the much smaller subset identified in the C24 vs. WT comparison, suggesting that the DE analysis of C24 mRNA-seq libraries was nevertheless able to identify *Smn*-specific changes in neuronal gene expression. The occurrence of loss of sensitivity as a consequence of the presence of non-neuronal cells in the isolated tissue samples was expected to have a more significant effect on ubiquitously expressed genes, while neuronal genes should be more efficiently detected in a C24 background. By performing a classification of the differentially expressed genes identified in each comparison regarding their described tissue specificity, we suggest that the major limitation in the transcriptome profiling of the C24 flies is a signal dampening effect arising from the presence of non-neuronal cells. Therefore, the use of this bulk approach in similar contexts of cell-type specific up or down-regulation of a target gene should be considered with caution when approaches to isolate the target cell population are not an option. However, our results suggest that the increased sensitivity in the identification of cell-type specific genes afforded by these models may still justify the combined use of tissue specific down-regulation and compound tissue libraries in specific cases.

While assessing the quality of our sequencing dataset, we observed a bias in the nucleotide frequencies of the first 10 nucleotides of the 5′ ends of all reads. This bias has been attributed to the use of random hexamer primers in the preparation of mRNA-seq libraries (Hansen et al., 2010). Therefore, we investigated what would be the impact of filtering out these nucleotides at the level of library size, rate of mapped reads and percentage of reads mapped to protein coding genes. While this manuscript was in review, Del Fabbro et al. (2013), reported that trimming 5′ ends of RNA-seq reads resulted in a decrease of library size and in the increase of the number of mapped reads. These results confirm our assessment of the effect of trimming 5′ ends of all reads. Moreover, and more interestingly our results showed the increase of reads that mapped to protein coding features, with the more prominent effect on genes with medium levels of expression. Given that the quality scores are estimated by an algorithm that analyses image data, this suggests that the measure of quality scores in the trimmed portion of reads has been overestimated and that trimming 5′ ends that suffer from a distortion nucleotide frequencies may result in a decrease of the effect of systematic technical errors, ensuring a more accurate measurement of gene expression. Indeed, after testing for DE, although we found a large overlap between both analysis, we observed a decrease in the number of differentially expressed genes, most likely due to a decrease in the false discovery rate.

In conclusion, we have observed that applying a small set of key genes allowed us to eliminate mRNA-seq libraries with a high level of contamination from non-CNS tissues, purging the dataset from libraries that would otherwise completely impair the identification of differentially expressed genes between the CNS transcriptome. Moreover, we address the limitations associated with the use of complex tissue samples to profile gene expression changes caused by a cell-type specific down-regulation of a gene of interest. Our results suggest that although the magnitude of detected changes—in particular for ubiquitously expressed genes—may be significantly masked by the detection of transcripts from other, non-targeted cell types, the ability to identify changes in genes that are specifically expressed in the targeted cell type is still maintained. Therefore, the combined use of models displaying tissue specific and somatic down-regulation of a gene of interest may provide complementary information. Finally, we demonstrate that trimming the 5′ end portion of mRNA-seq reads when significant biases in nucleotide frequencies are detected during quality control analysis, can increase the coverage of protein coding genes, causing a change in the number of DE genes, mostly likely to a decrease of incurred sampling bias.

## Conflict of interest statement

The authors declare that the research was conducted in the absence of any commercial or financial relationships that could be construed as a potential conflict of interest.
