# Supplementary material for: Quality assessment and control of tissue specific RNA-seq libraries of Drosophila transgenic RNAi models
Source: Front Genet. 2014 Mar 5;5:43. doi: 10.3389/fgene.2014.00043 (PMC3942661; doi:10.3389/fgene.2014.00043)
Supplement: Supplementary file 3 [file DataSheet3.DOCX]

**Supplementary Table 3| Primers and Probes to assess Smn expression by qPCR**

Two primers and Zen^TM^ double quenched probes sets were designed using PrimerQuest primer design tool provided by Integrated DNA Technologies, Inc. (Coralville, IA, USA) and synthesized by Integrated DNA Technologies, Inc., otherwise noted. Primers for Smn-A3 and Smn-A5 assay were design and synthesized by TaKaRa Bio Inc. (Shiga, Japan)

Note: Number front of Forward and Reverse primer sequence is starting position of each gene: TBP (NM_079081.3), and Smn (NM_079388)

TBP

Forward 1075-GCT ACG AGC CTG AGC TAT TT

Reverse 1171-CCA GTG AGC ACC ACC TTT

Probe /56-FAM/AAT CGT GCT /ZEN/CCT CAT CTT CGT GTC C/3IABkFQ/

Smn-A3

Forward 277- CTACCAGCCCGGAACCAGTATC

Reverse 407- CAAATAGCGGAGTACGCAGGTG

Probe /56-FAM/AGG GCG CTG /ZEN/TGG TGT CTA TTA ACG /3IABkFQ/
