# Supplementary material for: Quality assessment and control of tissue specific RNA-seq libraries of Drosophila transgenic RNAi models
Source: Front Genet. 2014 Mar 5;5:43. doi: 10.3389/fgene.2014.00043 (PMC3942661; doi:10.3389/fgene.2014.00043)
Supplement: Supplementary file 6 [file Presentation1.PDF]

Supplementary FILE 1

#########Bioinformatic analysis commands###########################

##
Alignment using BWA
##

bwa aln -n 1 -t 4 D_m_bwa_is [Read_1.fastq_TABLE_filtered.txt.fastq] > [Read_1.fastq_TABLE_filtered.txt.fastq.sai]

bwa sampe -n 1 -N 1 D_m_bwa_is [Read_1.fastq_TABLE_filtered.txt.fastq.sai] [Read_2.fastq_TABLE_filtered.txt.fastq.sai] [Read_1.fastq_TABLE_filtered.txt.fastq]   [Read_2.fastq_TABLE_filtered.txt.fastq] > [pair_sam] &

##
SAMTOOLS : for duplicate filtering : this was applied to sam output obtained from BWA and to accepted_hits.bam generated by Tophat2
##

samtools view -bS -o [pair.bam] [pair_sam] #convert sam to bam

samtools sort [pair.bam] [pair_sorted.bam] # Sort by chromossome

samtools rmdup [pair_sorted.bam] [pair_sorted_filtered.bam] #Remove duplicates

samtools sort -n [pair_sorted_filtered.bam] [pair_sorted_filtered_byread.bam] #Sort by read

samtools view -h -o [pair_sorted_filtered_byread.sam] [pair_sorted_filtered_byread.bam] #Convert back to .sam

##
RNA-seq read counts per gene using htseq-count
##

htseq-count -a 10 -s no [pair_sorted_filtered_byread.sam] Drosophila_melanogaster.BDGP5.66.gtf > [gene_count_union]

```
############################################################
Alignment using TopHat2

############################################################

tophat2  --num-threads 2 -r 200 -g 1 -G /Drosophila_melanogaster.BDGP5.66.gtf
-o _tophat_no_novel --no-novel-juncs Bowtie2Index_Matching_gtf/D_M
[Sample_R1_TABLE_filtered.txt.fastq] Sample_R2_TABLE_filtered.txt.fastq]
```
