# Supplementary material for: Quality assessment and control of tissue specific RNA-seq libraries of Drosophila transgenic RNAi models
Source: Front Genet. 2014 Mar 5;5:43. doi: 10.3389/fgene.2014.00043 (PMC3942661; doi:10.3389/fgene.2014.00043)

Supplementary Figure 1- Scatterplot showing the relation between the cDNA input of mRNA-seq libraries and the proportion of duplicates that was observed. x-axis (cDNA input in ng), y-axis % of duplicates in the mRNA-seq library

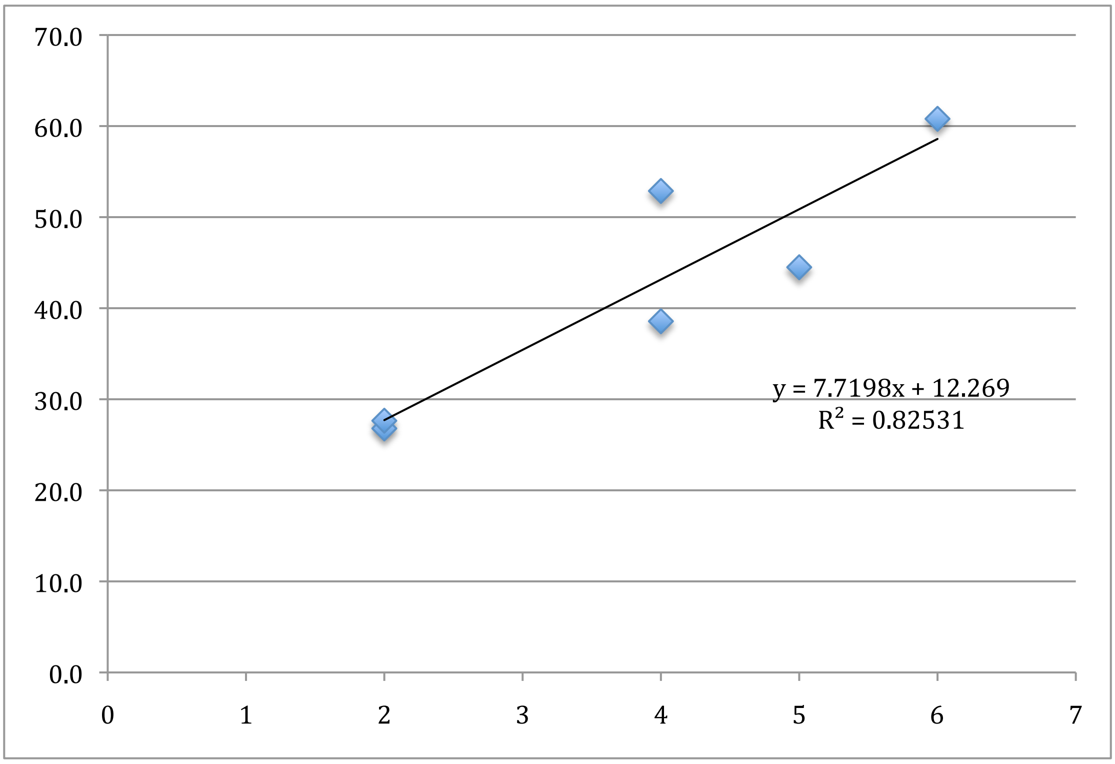

Supplement: Supplementary file 7 [file Presentation2.PDF]
